# Genetics, Recombination and Clinical Features of Human Rhinovirus Species C (HRV-C) Infections; Interactions of HRV-C with Other Respiratory Viruses

**DOI:** 10.1371/journal.pone.0008518

**Published:** 2009-12-30

**Authors:** Anne Wisdom, Aldona E. Kutkowska, E. Carol McWilliam Leitch, Eleanor Gaunt, Kate Templeton, Heli Harvala, Peter Simmonds

**Affiliations:** 1 Centre for Infectious Diseases, University of Edinburgh, Summerhall, Edinburgh, United Kingdom; 2 Specialist Virology Centre, Royal Infirmary of Edinburgh, Edinburgh, United Kingdom; Saint Louis University School of Medicine, United States of America

## Abstract

To estimate the frequency, molecular epidemiological and clinical associations of infection with the newly described species C variants of human rhinoviruses (HRV), 3243 diagnostic respiratory samples referred for diagnostic testing in Edinburgh were screened using a VP4-encoding region-based selective polymerase chain reaction (PCR) for HRV-C along with parallel PCR testing for 13 other respiratory viruses. HRV-C was the third most frequently detected behind respiratory syncytial virus (RSV) and adenovirus, with 141 infection episodes detected among 1885 subjects over 13 months (7.5%). Infections predominantly targeted the very young (median age 6–12 months; 80% of infections in those <2 years), occurred throughout the year but with peak incidence in early winter months. HRV-C was detected significantly more frequently among subjects with lower (LRT) and upper respiratory tract (URT) disease than controls without respiratory symptoms; HRV-C mono-infections were the second most frequently detected virus (behind RSV) in both disease presentations (6.9% and 7.8% of all cases respectively). HRV variants were classified by VP4/VP2 sequencing into 39 genotypically defined types, increasing the current total worldwide to 60. Through sequence comparisons of the 5′untranslated region (5′UTR), the majority grouped with species A (n = 96; 68%, described as HRV-Ca), the remainder forming a phylogenetically distinct 5′UTR group (HRV-Cc). Multiple and bidirectional recombination events between HRV-Ca and HRV-Cc variants and with HRV species A represents the most parsimonious explanation for their interspersed phylogeny relationships in the VP4/VP2-encoding region. No difference in age distribution, seasonality or disease associations was identified between HRV-Ca and HRV-Cc variants. HRV-C-infected subjects showed markedly reduced detection frequencies of RSV and other respiratory viruses, providing evidence for a major interfering effect of HRV-C on susceptibility to other respiratory virus infections. HRV-C's disease associations, its prevalence and evidence for interfering effects on other respiratory viruses mandates incorporation of rhinoviruses into future diagnostic virology screening.

## Introduction

Human rhinoviruses (HRVs) are members of the recently expanded *Enterovirus* genus within the family *Picornaviridae* [1]. Their genome organisation and structure are typical for picornaviruses, possessing a 7200 base single stranded RNA genome and a non-enveloped virion of approximately 30 nm in diameter. Human rhinoviruses were originally classifiable into two species, A and B with likely distinct evolutionary separate origins within the *Enterovirus* genus. However, they both share a primary tropism for the respiratory tract and species B and most species A variants use the intracellular adhesion molecule-1 receptor for entry [2]. Being acid-labile, HRVs do not colonise the gut, and are most commonly transmitted by the respiratory-salivary route, both by person-to-person contact and airborne transmission. In temperate countries, infections occur primarily in two peaks, the first between April and May and the second between September and October [3], [4].

Recent wider use of molecular detection methods in viral diagnostic screening has revealed the quite unexpected existence of a further species of human rhinoviruses [5]–[11]. These species C rhinoviruses (HRV-Cs) have been shown to be highly prevalent, with initial studies demonstrating frequent detection of this species in association with bronchiolitis and other lower respiratory tract disease [9], [12]–[14], in asthma exacerbations [9], [14]–[16] and otitis media [17].

HRVs show remarkable genetic and antigenic heterogeneity, with 74 and 25 serotypes being classified by cross-neutralisation assays in species A and B viruses respectively. Although species C variants have not yet been isolated or cultured *in vitro* and therefore cannot be formally assigned into separate serotypes, existing sequence data reveals perhaps even greater genetic heterogeneity than found within HRV-A and HRV-B. The large number of distinct genetic lineages identifiable by sequence comparisons in the VP4/VP2 gene region may thus correspond to different serotypes of HRV-C [13], [18], [19]. Furthermore, sequence divergence in VP1 and other external regions of the capsid is actually greater than between different HRV-A or –B serotypes, implying the existence of major antigenic differences that influence immunological cross-protection in this newly discovered species. An additional aspect of HRV-C genetic diversity that has caused confusion in previous attempts at HRV species identification and typing is the occurrence of recombination between HRV-A and HRV-A in the 5′ untranslated region (5′UTR). While species A and B rhinoviruses have phylogenetically distinct sequences in this region, those from the majority of HRV-C variants resemble those of species A [19]–[21], while the remainder form a phylogenetically separate group distinct from HRV-A and HRV-B and other enterovirus species. Initial data however shows no differences in clinical presentations between HRV-C variants with A and C 5′UTR-like sequences (described as HRV-Ca and HRV-Cc respectively [19]; this nomenclature will be followed in the current study).

In the current study we have developed a selective VP4 gene PCR-based amplification method for specific detection of HRV-C in respiratory specimens. This has enabled us to screen and clinically characterise infections occurring over a whole calendar year (13 months) in a study group of more than 1800 subjects. Parallel screening for other human respiratory viruses over the same period allowed us to directly compare their epidemiologies and association with specific disease presentations.

## Results

### Development of a Method for Selective Screening of HRV-C

The intention of the study was the investigation of the incidence, genetic variability and clinical presentation of HRV-C collected over a whole calendar year. Non-selective amplification of all HRV (and HEV) species using previously developed 5′UTR-based PCR methods would have identified an extremely large number of non-HRV-C positive samples that would preclude effective screening using pools. To overcome this, we modified the VP4/VP2-encoding region PCR previously used for genetic characterisation and (sero)type identification of HRV to enable selective amplification of HRV-C. The inner antisense primer was repositioned to a more variable position in the VP4 gene between species A, B and C sequences to enable selective hybridisation and strand extension of HRV-C sequences. The sensitivity and specificity of the selective PCR was evaluated using samples previously identified as HRV-A, -B and –C variants in a previous study [18]. All 26 species C samples were positive, along with 1 from 63 species A and 1 from 8 species B samples in the assay (sensitivity 100%; 98% specificity). Since all positive samples were to be sequenced in the more variable VP4/VP2-encoding region, this minor degree of non-specificity would not influence our identification of HRV-C positive samples for the study.

### Detection of HRV-C in Respiratory Samples

Excluding those samples from September 2006 and February 2007 already tested as controls for assay validation, the assay was used for screening the remaining samples between September 2006 and September 2007. These comprised a total of 2787 samples collected from 1540 study subjects initially combined into pools of 10 and with positive pools split and re-tested as previously described [22].

A total of 162 samples were positive on initial screening. On nucleotide sequencing of the amplified VP4/VP2 gene region, 144 samples were identified as belonging to species C, the remainder comprising HRV-A (n = 15), HRV-B (n = 2) and human enterovirus species B (n = 1). The selective primers were therefore moderately effective at selective amplification of species C variants with an overall specificity of 89% in a screened population (where HRV species A predominates; [18]). Screening data from the selective primers was combined with results from our previous screening of 456 samples collected in September, 2006 and February, 2007 [18] to yield a combined sample and study group of 175 HRV-C positive samples originating from 130 different individuals, to which further results will refer.

**Figure 1:**
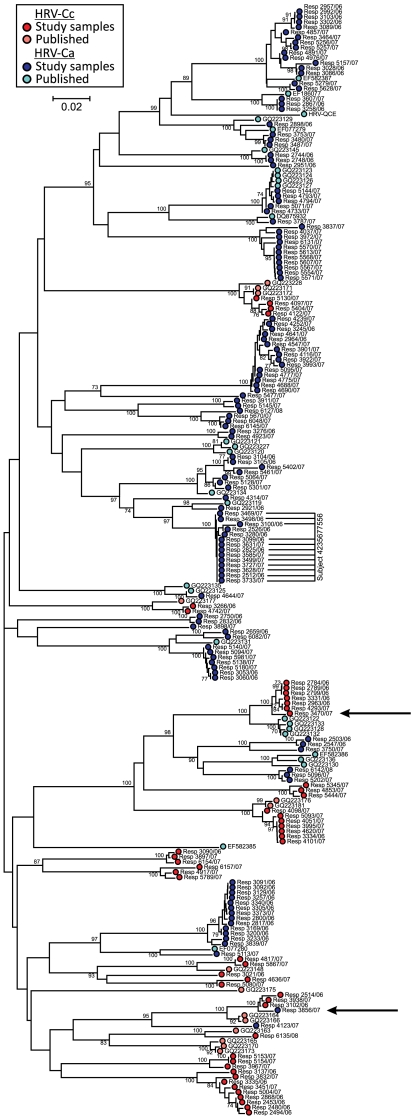
Phylogeny of VP4/2 gene sequences amplified from study subjects and comparison with corresponding full length sequences of HRV-C and other samples where 5′UTR groups had been previously assigned [**19**]. Symbols identify HRV-Cc (*ie.* with species C-like 5′UTR sequences) or HRV-Ca (species A-like) as described in the key. The neighbour-joining tree was constructed using maximum composite likelihood distances estimated between sequences in the amplified region (positions 629–1063 numbered according to the HRV-B14 reference sequence (NC_001490). Data was bootstrap re-sampled 100 times to assess robustness of branches; values of 70% or greater shown. Sequences showing incompatibilities between 5′UTR and phylogenetic grouping in VP4/VP2 suggestive of recombination are arrowed.

Sequences amplified and sequenced in the VP4/VP2-encoding region were compared with those obtained in previous studies (sequence download from GenBank in August, 2009). Amongst the latter previously deposited sequences, a total of 54 putative HRV-C (geno)types could be assigned using previously described criteria (VP4/VP2 gene phylogeny and a pairwise distance threshold of 0.1; [18]). Through the same comparative method, study samples comprised 39 types (Fig. 1), of which 6 were distinct from previously deposited HRV-C sequences, producing a combined total of 60.

Frequencies of re-infection and persistence of HRV-C among subjects who contributed more than one positive sample over the study period were estimated by sequence comparisons in the VP4/VP2-encoding region. For the 13 subjects who were repeatedly positive for HRV-C in the same calendar month, VP4/VP2 gene sequences were similar or identical, while those with positive samples separated by one month or longer were invariably infected with different HRV-C types. The close concordance between sample timing and evidence for re-infection allowed us to identify separate infection episodes in 11 of the subjects. The only exception to this correlation was a long-term hospitalised patient with acute myeloid leukaemia, who was regularly screened through the 13 month study period and from whom a total of 12 positive samples (from 19 collected) were obtained. The close sequence similarity of HRV-C variants over this period (marked in Fig. 1, subject ID 4235677556) indicated long-term persistent HRV-C infection in the context of likely severe immunosuppression.

### HRV-C Recombination

The 5′UTR group of each of the HRV-C positive samples in the study was determined by amplification and sequencing of a region between 280–370 in the 5′UTR. This analysis was assisted by inclusion of those published HRV-C for which VP4/VP2 gene sequences and 5′UTR groups had been determined (including the 9 available complete genome sequences of HRV-C; data not shown). The majority of HRV-C variants characterised in the current study from separate infection episodes (96 from 141; 68%) contained species A-like 5′UTR sequences, termed HRV-Ca [19]. Clades and HRV-C types with different 5′UTR groups (HRV-Ca and HRV-Cc) were interspersed within the VP4/VP2 gene tree (Fig. 1), implying the existence of multiple recombination events in the past diversification of HRV-C. In general, all variants of the same (geno)type assigned in the VP4/VP2-encoding region contained the same 5′UTR group. There were however, two exceptions, the HRV-Ca samples R3856/07 clustered closely with HRV-Cc variants (Fig. 1) and there was a split of one of the genotypes into HRV-Ca (published sequences from China) HRV-Cc (7 samples amplified from 5 different study subject from Edinburgh). These observations imply ongoing, bi-directional and frequently recent recombination events between rhinoviruses with different 5′UTR groups.

### Epidemiological and Clinical Associations of HRV-C

**Figure 2:**
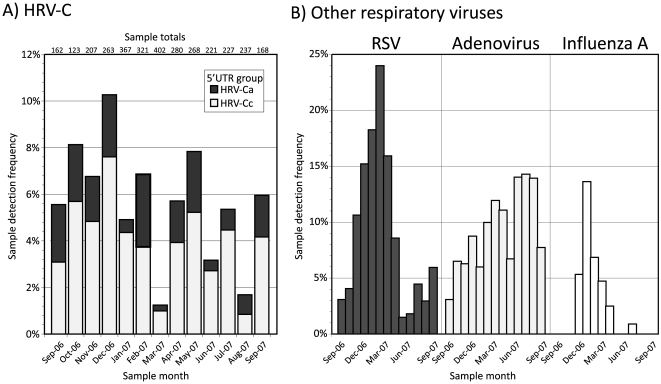
Seasonal distribution of (A) HRV-C positive samples and (B) those positive for other common respiratory viruses. The y-axis depicts the proportion of samples positive from the total in each category (shown above each bar in part A). Frequencies of HRV-C detection have been subdivided into HRV-Ca and HRV-Cc subgroups.

**Figure 3:**
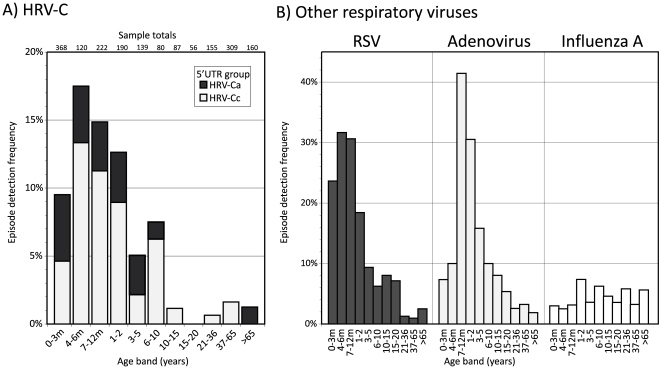
Age distribution of (A) HRV-C infected subjects and (B) those infected with other common respiratory viruses. The y-axis depicts the proportion of subjects positive from the total in each category. Frequencies of HRV-C detection have been subdivided into HRV-Ca and HRV-Cc subgroups.

Epidemiological and clinical information on the study samples and subjects retained through the anonymisation process was used to compare seasonal and age distribution of HRV-C infections with those of other common respiratory virus infections (Figs. 2, 3). In contrast to RSV and influenza A virus which showed pronounced increases in incidence in winter months, HRV-C infections occurred year round with similar incidence in each calendar month investigated in the current study (Fig. 2A), more comparable to adenovirus. In contrast, HRV-C resembled RSV closely in targeting young children and infants, with peak incidence of both viruses occurring in the 4–6 month age range, with a younger age distribution than adenovirus infections and distinct from the wide age range of influenza A virus (Fig. 3). 85% of HRV-C infections were observed in children younger than 2 years of age. No systematic difference was evident between HRV-Ca and HRV-Cc in their seasonal distribution or age range of infection.

**Figure 4:**
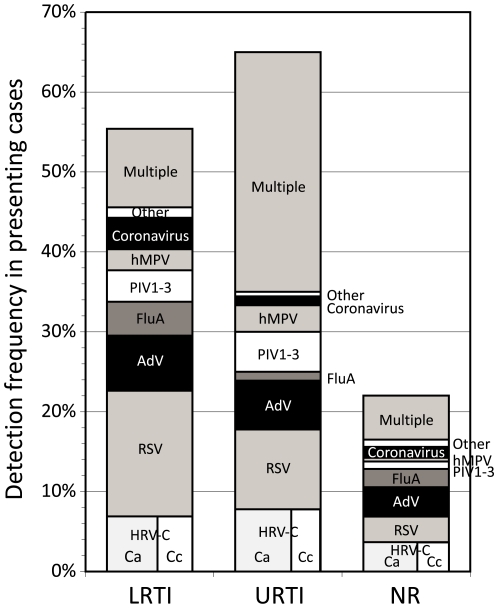
Detection frequency of HRV-C and other respiratory viruses among subjects presenting with symptoms or diagnoses of LRTIs, URTIs and those with no relevant (NR) respiratory symptoms. For the purposes of this analysis, cough was considered a symptom of URTI.

Detection frequencies of HRV-C and other respiratory viruses among subjects presenting with LRTIs, URTIs and those without respiratory symptoms were compared (Fig. 4). Subjects compared were those presenting acutely and excluded those with underlying immunosuppression or severe underlying disease (see Methods for criteria to assign disease categories). One or more respiratory virus was detected in 55% of subjects with LRTIs, 65% in URTIs and 22% in those with no relevant respiratory or other symptoms/diagnoses. Dividing these groups, HRV-C was the only virus detected in 6.9% of LRTI cases and in 7.8% in URTIs, higher than in the non-respiratory disease controls (3.7%) and second only to RSV in detection frequency (15.7% and 10.0% respectively). HRV-C was twice as frequently detected in subjects with respiratory disease than in those without relevant symptoms (*p* = 0.009 by Chi-squared test). HRV-C was detected in children in paediatric intensive care units (12%, 14 patients), mostly with pneumonia and without any other cause identified, in neonatal intensive care (11%, 4 patients). There was no significant difference between disease categories in the proportion of HRV-Ca and HRV-Cc variants.

### Virus Interference

**Figure 5:**
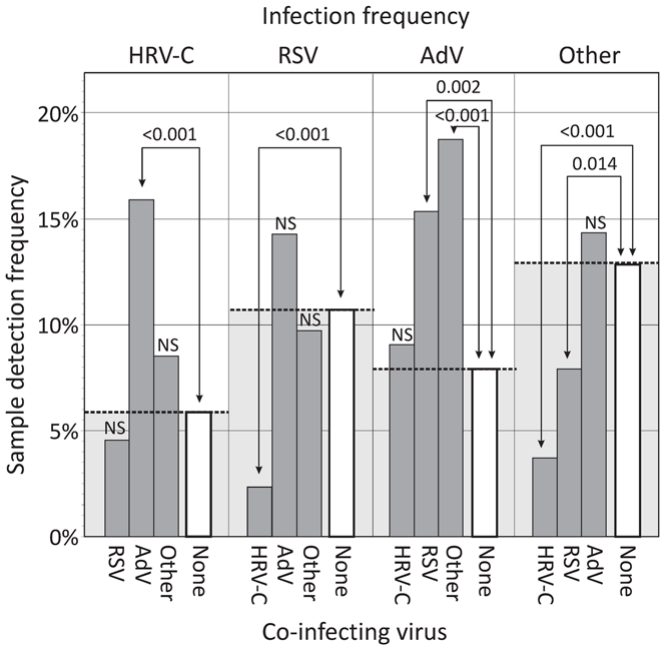
Analysis of potential virus interference through comparison of mono- and co-infection frequencies. Each of the three most frequently detected respiratory viruses (HRV-C, RSV and AdV) and a combined group of all others (influenza A and B, PIV1-3, hMPV, coronaviruses, HBoV, HPeV) were separately analysed (x-axis, top). For each, mono-infection frequencies (unfilled bars) were compared with those where HRV-C, RSV, AdV and other respiratory viruses were co-detected (indicated on bottom x-axis, grey filled boxes). The existence of statistically significant differences in frequencies was determined by chi-squared test; NS: not significant.

Evidence for significant interactions between respiratory viruses was obtained through comparisons of detection frequencies in mono- and doubly-infected individuals (Fig. 5). HRV-C was nearly three times more commonly detected in those co-infected with adenovirus (16.0%) than in mono-infected subjects (5.8%; *p*<0.001). Examples of negative interactions were additionally observed. Most strikingly, HRV-C had a highly significant effect on detection frequencies of RSV and other respiratory viruses. RSV mono-infection was found in 10.7% samples but virtually absent among HRV-C infected subjects (2.4%, *p*<0.001). For other respiratory viruses (excluding AdV), a 3.5-fold reduction in their infection frequency was observed in HRV-C co-infected subjects (12.9% to 3.7%; *p*<0.001). This reduction was greater than the effect of RSV co-infection on their detection frequency (<2-fold). In contrast, RSV and the group of other respiratory viruses did not significantly influence the likelihood of HRV-C detection; study subjects showed a 5.8% HRV-C mono-infection frequency compared to 4.6% and 8.6% in those co-infected with RSV and other respiratory viruses respectively (*p*>0.05). Together these observations indicate that it is HRV-C that is inhibiting RSV and other respiratory infections rather than the converse.

## Discussion

### Detection Frequency and Type Identification of HRV-C

This study represents one of the first large scale investigations of the epidemiology and clinical impact focussed on the newly discovered species C rhinoviruses, and provides evidence for its significant role in childhood respiratory disease. HRV-C was the third most prevalent virus in the study population with 141 infection episodes detected among 1885 subjects over a 13 month period (7.5%), with only RSV (14.2%) and adenovirus (11.5%) being detected more frequently. Through genetic comparisons of the VP4/VP2-encoding region, HRV-C infections were found to be almost invariably acute/resolving with no evidence for persistence among multiply sampled individuals with the single exception of an immunocompromised child with AML. Where re-infection did occur, it was invariably by another HRV-C type, consistent with the hypothesis that species C types that have been assigned on the basis of VP4/VP2 gene sequence divergence, might nevertheless be antigenically distinct and capable of infection in those exposed previously to heterologous types. These findings are consistent with previous observations for a 10–21 day period of virus shedding of HRV in immunocompetent individuals [23]–[26]. Given the high incidence of HRV infections and ongoing exposure to other circulating (sero)types, molecular characterisation is required to clearly document HRV persistence [27].

Contributing to the high incidence of HRV-C is its genetic (and likely antigenic) diversity, evident among the variants detected in the current study and through analysis of published sequences in the VP4/VP2-encoding region where a total of 60 types could be provisionally assigned. Although nucleotide sequence divergence in this region can only be used indirectly to infer antigenic variability in other parts of the genome, the 10% pairwise distance threshold in VP4/VP2-encoding region matched the divergence that effectively categorises HRV species A and B into their 74 and 25 constituent serotypes [18]. Sequence comparisons in the VP4/VP2-encoding region thus remain a useful provisional assignment tool until sequences from the whole capsid-encoding region become available.

### HRV-C Recombination

Through sequence comparisons in the 5′UTR of the samples identified as positive on VP4 gene screening, it was possible to classify the 175 positive samples into two groups, those with a 5′UTR sequence similar to species A rhinoviruses described as HRV-Ca [19] and those clustering separately from species A and B (and from human enteroviruses) described as HRV-Cc. The complete genome sequence N10 [19] falls into the HRV-Cc group while the eight others available, including the species C prototype strain QPM possess 5′UTR sequences groups within HRV species A sequences (HRV-Ca). Apart from the complete genome sequences, published sequence data from both the 5′UTR and VP4/VP2 gene regions is only available from 34 Chinese species C strains [19]. These were classified into 20 HRV-Ca and 14 HRV-Cc variants. Other studies from South Korea [21] and Italy [26] separated species C variants into HRV-Ca and HRV-Cc groups, although VP4/VP2 gene sequences were not deposited onto GenBank/EMBL and could not be included in the analysis in Fig. 1.

The multiple interspersed phylogenetic positions of VP4/VP2-encoding sequences from HRV-Ca and HRV-Cc variants is consistent with multiple inter-species recombination events in the evolutionary history of human rhinoviruses. We do not know and likely cannot reconstruct the actual evolutionary steps that created the complex phylogeny relationships in this genomic region, or the nature of the ancestors of currently circulating species A and C rhinoviruses. Nevertheless the most parsimonious explanation for the scattering of HRV-Cc and HRV-Ca variants in the VP4/VP2 tree is a series of recombination events early in the diversification of the ancestor of HRV-C into the current large number of types that now exist. Had there been only one recombination event between species A and C, the descendants of the original recombinant (with its more recent evolutionary origin relative to rhinoviruses circulating at that time) would be expected to have remained more closely related to each other (monophyletic) in both 5′UTR and VP4/VP2 gene regions (and elsewhere in the genome). This is clearly not the case in the VP4/VP2 gene region (Fig. 1) or in the 5′UTR; published HRV-Ca variants (those of complete genome sequences and from China) fall into at least two separate groups in the latter region [19]. The occurrence of multiple recombination events is consistent with recent observations for differences in the recombination breakpoints between different HRV-Ca and HRV-Cc variants [19].

The observation that variants within a species C type usually had the same 5′UTR group (Fig. 1) suggests that most recombination occurred early in the diversification of HRV-C. However, a variant in the current study differed in 5′UTR group from other members of the same type (the HRV-Ca strain R3856/07 clustered with HRV-Cc variants). Although these anomalous results have been verified by independent re-extraction and re-amplification of the sample and other members of the clades into which it grouped, it remains formally possible that the study subject was co-infected with two different rhinovirus variants. PCR assays using 5′UTR and VP4/VP2 gene primers may have amplified a different (species A-like) 5′UTR sequence from that possessed by the species C virus sequenced in the VP4 gene. Notwithstanding this, another recent (intra-typic) recombination event could be inferred between a cluster of closely related HRV-Cc variants from the current study and several published HRV-Ca sequences from China that are of the same species C type (Fig. 1). Given this evidence for frequent and likely ongoing recombination, it remains difficult to explain why there are no known species A rhinovirus recombinants with species C 5′UTR sequences; perhaps ongoing recombination events are restricted to those between HRV-Ca and HRV-Cc.

In many respects, the phylogeny relationships observed in rhinoviruses in 5′UTR and coding regions resemble those of human enteroviruses. Santti *et al.* [28] showed that 5′UTR sequences from HEV-A and -B were similarly interspersed in this region, as were sequences from species C and D, implying a similar process of frequent interspecies recombination. Whether inter-species recombination in rhinovirus species C (or indeed among enteroviruses) confers an evolutionary advantage remains unclear. Observations for its (likely) multiple occurrence in species C and the absence of species A rhinoviruses with group C 5′UTR sequences suggests that possession of group A 5′UTR may be advantageous. However, no evidence was gained in this study for any distinct epidemiological features or in their clinical presentations that might originate from hypothesised fitness differences between HRV-Ca and HRV-Cc groups.

### Clinical Presentations of HRV-C

Comprehensive screening for HRV-C over a whole calendar year enabled seasonality of infections, target groups and clinical associations of HRV-C infections to be compared with those of other respiratory viruses. The large number of HRV-C infection episodes detected over this period allowed a number of relatively robust conclusions to be drawn. Firstly, the study identifies young children and infants as the main target group for HRV-C infection among predominantly hospitalised individuals, with an age profile comparable to that of RSV. For both viruses, over 80% of infections were observed in those less than 2 years of age (Fig. 3), higher than in AdV (77%), influenza A virus (44%) and other respiratory viruses (63%). The targeting of very young children was comparable to that observed for HRV species A (80%; [18]) and in previous investigations of all three HRV species [13], [14], [29], [30]. HRV-C similarly resembled other rhinovirus species in its seasonal distribution (Fig. 2), circulating throughout the year as reported for other rhinoviruses [3], [13], [25], [29], [31]. It did not however show the typically higher incidence in autumn found in other rhinoviruses (and human enteroviruses), and actually showed higher infection frequencies in winter months. However, a longer study period would be required to discount the effect of year-on-year fluctuations in the incidence of species C.

It is increasingly recognised that HRVs are collectively underestimated as a cause of significant respiratory illness [27], [32]. The frequent detection of HRV-C mono-infection in a substantial proportion of subjects presenting with LRTIs and URTIs (Fig. 4) is concordant with recent PCR-based studies [13]–[17], [29], [33], [34] that demonstrate a numerically significant role for HRV-C and other rhinovirus species in severe respiratory disease requiring hospitalisation in children and exacerbation of asthma. Detection frequencies of 7–8% for HRV-C in subjects with URTIs or LRTIs was significantly higher than in those presenting without respiratory disease in the current study (3.7%), although this approximate two-fold difference in frequency was less than observed between these categories for RSV and hMPV (4- and 6.5-fold), parainfluenza viruses (5-fold), but greater than for adenoviruses and coronaviruses (1.5- and 1.8-fold) and other viruses (HBoV/HPeV; equal frequencies). To our knowledge, there is no published information on the rate of asymptomatic infections with species C rhinoviruses in hospitalised patients or in the general community. However, total HRV detection frequencies determined by PCR have produced highly variable results (2–61%; reviewed in [27]), although averaging to 15% [35]. Taking this latter average estimate, and assuming that species C variants constitute a quarter or a third of all HRV detections (as previously found in a subset of the current study population; [18]), then the 4%–5% predicted prevalence matches closely to that of the control group in the current study. The increased frequency in the LRTI and URTI groups is therefore consistent with its proposed aetiological role in respiratory disease among the predominantly hospitalised study subjects in the current study. Further prospectively collected information on disease severity and symptoms, along with viral load information on study subjects and controls are, however, required to further substantiate the findings reported here.

### HRV and Co-Infection Frequency

In the current study, all samples were screened for an extended range of human respiratory viruses (influenza A and B viruses, RSV, AdV and PIV1-3, hMPV, HBoV, HPeV and coronaviruses). This allowed a detailed investigation of frequencies of virus co-infections among subjects presenting with respiratory illnesses and potential interactions (positive and negative) between them. The most striking observation was the evidence for interference of RSV and other respiratory viruses (except adenovirus) by HRV-C. For example, the 10.7% detection frequency of RSV among single-infected individuals was markedly reduced among those co-infected with HRV-C (2.4%). A comparable reduction was observed in the “other virus” category (predominantly parainfluenza and coronaviruses with a 3.5-fold difference). RSV showed a more modest effect on detection frequencies in this latter category (less than two-fold reduction). The further observation that the presence of RSV or other viruses did not conversely influence HRV-C detection frequencies is consistent with HRV-C interfering with susceptibility to infection with other RNA viruses rather than the opposite. Negative interactions in detection frequency and viral loads comparable to those observed between these RNA virus groups have been previously described between different pairs of respiratory viruses, and report significant interfering effects of HRV (all species) on RSV and a range of other RNA viruses [36], [37]. In contrast to the latter study, we additionally found positive interactions, with significantly higher detection frequencies of RSV and other RNA viruses in AdV-positive samples.

Interpreting the mechanisms underlying these observations of virus interference is complex. One unknown factor in this and previous studies that may significantly contribute to the outcome of a virus/virus interaction is the order of acquisition of infections. For example, it has been hypothesised that the strong interferon (IFN) response in the respiratory tract induced by HRV infection may create an environment hostile to infection with other viruses, such as RSV [36]. Even though RSV, through expression of NS-1, can prevent IFN induction on infection of a cell [38], this countermeasure may be largely ineffective in a respiratory tract already induced into an antiviral state by prior infection with HRV-C. If, however, RSV infected the respiratory tract first, then this would have no effect on the subsequent susceptibility of the individual to HRV. The rapid and highly cytopathic replication cycle of rhinoviruses (and enteroviruses) that seems designed to infect and escape from cells before IFN-mediated responses become effective, may indeed have a more general interfering effect on more sensitive RNA (and DNA viruses) that infect the respiratory tract. Like RSV, successful colonisation of the respiratory tract by coronaviruses, influenza and parainfluenza viruses is dependent on a wide variety of evolved mechanisms to evade intracellular defences [39], [40] that are ineffective in pre-sensitised cells induced by IFN into an antiviral state.

The effect of the order in which infections are acquired may additionally influence the outcomes of co-infections with adenoviruses. AdVs express a plethora of evasion molecules that substantially influences both the intracellular environment of the cell it infects and also has a broader paracrine effect on cytokine production in the respiratory tract and induction of local immunity [41]. The frequent long term persistence of AdV infections suggests that a more permissive environment for infection and replication by other viruses may exist in the respiratory tract of AdV-infected individual, and underlie the increased detection frequencies of HRV-C, RSV and other respiratory viruses in co-infected subjects (Fig. 5).

In summary, this study provides evidence for the substantial clinical impact of HRV species C infections in young children, their associations with respiratory disease and interfering effects on susceptibility to other virus infections. Future studies should illuminate the extent to which these observations are specific to species C and which are shared with other rhinoviruses. The recent surge of interest in rhinoviruses following the unexpected discovery of species C will undoubtedly contribute to a major re-appraisal of the roles of all three species in paediatric respiratory disease.

## Materials and Methods

### Study Population and Samples

HRV-C was screened for in 2787 respiratory samples from 1540 patients (839 male, 675 female, 26 unknown) referred for respiratory virus screening to the Specialist Virology Centre (SVC), Royal Infirmary of Edinburgh between September 2006 and September 2007. The study incorporated previously collected data from 456 samples collected in September, 2006 and February, 2007 [18]. Samples comprised predominantly nasopharyngeal or tracheal aspirates or swabs (n = 1771), throat swabs (n = 619) and bronchoalveolar lavages (n = 155), and were routinely screened for the following respiratory viruses by PCR: adenovirus (AdV), influenza A and B, parainfluenza virus types 1–3 (PIV1-3) and respiratory syncytial virus (RSV) using previously described assays [42], [43]. Samples were further tested for human bocavirus (HBoV), human parechovirus (HPeV) [44], human metapneumovirus (hMPV) [45] and coronaviruses HKU1, OC43, 229E and NL63 (Gaunt *et al.,* manuscript in preparation). All but 94 (2.9%) of the tested samples were referred from hospital inpatients or from Accident and Emergency Departments. Lower respiratory tract infections (LRTIs) were identified in subjects presenting with the following symptoms or diagnoses: bronchiolitis, chest infections, pneumonia, respiratory failure, shortness of breath, apnoea and wheeze; upper respiratory tract infections (URTIs) with coryza, sore throat, tonsillitis and tracheitis. Cough was also classified as an URTI symptom, although we acknowledge its additional potential association with lower respiratory tract disease that leads to some potential misclassification of disease categories in the absence of other respiratory symptoms. Subjects with no relevant respiratory symptoms included the referral categories of vomiting, diarrhoea, trauma and elective cardiac surgery.

Ethical approval for anonymisation, archiving and screening of diagnostic specimens was obtained from the Lothian Regional Ethics Committee (08/S11/02/2). Information retained through anonymisation included age band, partial postcode, any recorded symptoms or clinical information, referral source, month of sample collection, and results of other virological testing of each sample.

### HRV-C Screening

RNA was extracted from clinical specimens as previously described [18]. Reverse transcription of RNA extracted from individual samples or pools of ten with random hexamers used the RT system (Promega, United Kingdom) as described previously [44]. Amplification of the HRV-C VP4/VP2-encoding region used previously described primers and reaction conditions [18], with a repositioned antisense inner primer ( ATA GTR ATT TGY TTD AGC CTA TCD GAV A ; 5′ base at position 888 numbered here and elsewhere according to the HRV species B reference sequence NC_001490) for selective amplification of HRV-C (see Results). Underlined bases were designed to mismatch species A or B (or both) sequences while being conserved within species C. Positive samples were subsequently amplified with the four original VP4/VP2 gene primers [18] and the amplicon directly sequenced. Sequences generated from this study were edited and aligned with published sequences using the Simmonics sequence editor v1.7 ([46], http://www.virus-evolution.org). Published sequences included the 9 complete genome sequences of HRV-C available on GenBank and all species C VP4(/VP2) gene sequences available on GenBank in August 2009. HRV-C types were putatively assigned by phylogenetic analysis and uncorrected pairwise distance measurements using the previously proposed 10% divergence threshold [18]. Phylogenetic trees were constructed by neighbour joining from 100 samplings of maximum-composite-likelihood (MCL) distances using the MEGA 4.0 software package [47] with pairwise deletion for missing data. VP4/VP2 sequences generated in this study have been assigned GenBank accession numbers GU294336-GU294480.

HRV-C positive samples identified on VP4/VP2 gene screening were amplified in the 5′UTR region for group assignment used a hemi-nested PCR with primer 178 (HCA AGY ACT TCT GTY WCC CCS G) used for both first and second round with the antisense primers 573 (GAA ACA CGG ACA CCC AAA GTA GT; outer) and 477 (TTA GCC RCA TTC AGG GGC CGG; inner) using previously described reaction conditions [18]. For a small number of samples that were unamplifiable by this method, SuperScript III (Invitrogen) was used for reverse transcription and first round PCR. HRV-C variants were assigned into species A and C 5′UTR groups by comparison with reference sequences of known group between positions 280 and 370.

### Statistical Analyses

Statistical tests were conducted using chi squared test with Yates' correction and 95% confidence intervals unless stated.
